# Open-source software utilization for zebrafish embryos behavior test

**DOI:** 10.1016/j.mocell.2025.100221

**Published:** 2025-04-25

**Authors:** Thilini Ranasinghe, Seon-Heui Cha

**Affiliations:** 1 Department of Marine Bio and Medical Sciences, Hanseo University, Seosan 31962, Republic of Korea; 2 Department of AquaLife Medicine, Hanseo University, Seosan 31962, Republic of Korea; 3 Institute for International Fisheries Science, Hanseo University, Seosan 31962, Republic of Korea

**Keywords:** Behavior, Neurology, Toxicology, Embryo, Zebrafish

## Abstract

This work described simple methods for measuring locomotive activity using open-source software, ImageJ1.54fFiji, and VirtualDub2. The significance of animal behavior is a mirror of brain activity, which can give information implicated with neurological diseases. Commercial behavioral analysis software frequently needs expertise and expenses high costs due to equip a specific instrument to use of software, thereby encouraging a trend toward open-source alternatives that are both accessible and effective. Here, we explained how to convert video format, measure movement, and produce useful locomotive parameters to aid in the assessment of zebrafish embryos. This method could be easily translated for use in other model systems. This methodology seeks to streamline behavioral quantification in research contexts, encouraging broader research aspects.

## INTRODUCTION

Behavior changes are used as indicators to explain the appearance of human diseases, such as behavioral disorders including oppositional defiant disorder, attention deficit hyperactivity disorder, and personality disorders (Sönmez and Kayaalp, 2018). Sudden disorientation, abnormal behavior, or distraction are diseases caused by altered mental status. Behavioral abnormalities are also used to predict the presence or absence of those diseases before computed tomography or histopathological examinations, as these are involved in behavioral changes (Mega et al., 1996, Phudphad et al., 2024, Sönmez and Kayaalp, 2018). Additionally, ocular diseases can a onset of behavioral abnormalities due to loss of object recognition ability and optic nerve damage caused by eye damage (Gorges et al., 2014).

There is no doubt that animal behavior is likely to be an expression of the brain activity of the animal, and observing animal behavior can provide information about neurotransmitters, neurons, and the entire brain mechanism (Simmons and Young, 2010, Stomp et al., 2021). Therefore, researchers interested in learning about the human and animal brains have become keener on behavioral symptoms. In particular, when using animal models, researchers are looking for new ways to trace and quantify behavioral changes that help find remedies for neurological-related diseases.

Zebrafish are an increasingly used model organism to study behavioral study relevant with neural basis due to their genetic homology to humans, and brain analysis is relatively easy (Kalueff et al., 2014). Although the brain structures are slightly different between zebrafish and mammals, they have similar structures that function in behavior, so they can perform behaviors similar to those of mammals, even though they are fish (Bailey et al., 2015, Kalueff et al., 2014). That is why many studies have used zebrafish to conduct behavioral analyses of brain diseases such as Parkinson's disease, Alzheimer’s disease, and chemical-induced neurotoxicity (Johnson et al., 2023, Ranasinghe et al., 2024, Yuan et al., 2022). For this reason, more experimental equipment and relevance software that can quantify the recorded behavior have been developed to record and track animal behavior.

However, the cost of building these instruments and software for behavioral analysis in research environments is becoming a risk factor and most of them need fluent users. Here, we provide a guide that how to manipulate the open-source software, ImageJ1.54fFiji (NIH, MD, USA), to measure zebrafish embryo (a powerful model organism for human disease) behavior.

## MAIN BODY

### Software Installation

ImageJ1.54fFiji version can be downloaded from the imagej.net website (https://imagej.net/software/fiji/downloads). Extra plugins needs for a operation should be downloaded by searching a plugin name in the search bar of the same website, or plugins can be updated by the help menu clicking in the ImageJ1.5fFiji window. VirtualDub2 software (SourceForge, CA, USA) for video format creation compatible with ImageJ1.54fFiji can be downloaded from SourceForge.net (https://sourceforge.net/projects/vdfiltermod/).

### Zebrafish Embryo Behavior Tracking and Measurement

#### Manual Tracking: Protocol 1, Use Manual Tracking Plugin

**Fig. 1:**
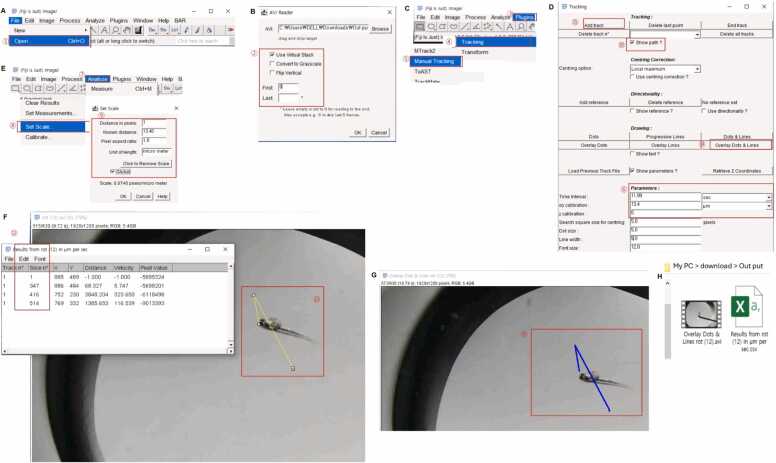
Method for locomotive activity test using ImageJ1.56fFiji “Manual Track” plugin, showing an example for manual tracking by touch response test and agitation challenge recorded videos. This method would be used to quantify the locomotive activity of behavior recorded videos as distance (movement traveled), velocity, and to generate videos with visible swim paths. (A) Path to open the video in Fiji ImageJ. (B) AVI Reader window box for selecting the stack range of the video to be opened, 1 stack = 0.02 s. (C) Path for opening the Manual Tracking plugin. (D) Tracking window box, assigning the time interval in sec, x/y calibration in the needed distance measuring unit relevant for 1 pixel of the video, and z calibration as 0 as the z-axis distance was not measured for the current experiment. (E) The main window for calculating distance in pixel from a video file’s scale. (F) and (G) Manual tracking in *.avi format video and automatically generated behavior measurements in *.csv format. (H) The output of behavior tracked video in *.avi format and behavior measurements in *.csv format. Red color numbering denotes the sequence of steps by which the method of manual tracking.

The method for tracking locomotor activity from a video can use the Manual Tracking plugin of ImageJ1.54fFiji (NIH) to generate a distance traveled, velocity, and visible swim path (Ranasinghe et al., 2024). First, open a *.avi format video file, and then AVI Reader window will pop up (Fig. 1A and B). Check the use of visual stack, and set the first stack to 0 and the last stack left empty (Fig. 1B), then the whole recorded video file could be opened as an image stack in ImageJ1.56fFiji. Thereafter, select the “Plugins > Tracking > Manual Tracking” menu (Fig. 1C), then the Tracking window will appear (Fig. 1D). In the Tracking window, put the video length in the time interval parameter; the time of the video length can be obtained in the top left corner of the video opened. To get a traveled length from a video file, put the scale bar length that obtained from the same magnification at which the video was recorded into the Main menu > Analyze > Set Scale to obtain the length for 1 pixel (x/y calibration, Fig. 1E), the x/y calibrated value put in x/y calibration parameter of the Tracking window (Fig.1D). The stack of video lengths can be found in the top left corner of the video opened as slice numbers (Fig. 1F). And select the Add track (Fig. 1D), to get the swim path together, check the Show path in the Tracking window (Fig. 1D). Thereafter, it should click on the video opened in ImageJ1.56fFiji, stack by stack from the beginning to the end of the video. Manual tracking will be marked in color (Fig. 1D). Select overlay dots and lines (Fig. 1D), and a tracked swim path video will be generated as a result (Fig. 1D). The completed moving track of the video, as shown in Figure 1H, could be saved in a desired file folder for later use. Finally, the locomotive activity could be obtained as the velocity traveled and distance of embryos in a *.csv format file (Fig. 1H).

#### Manual Tracking: Protocol 2, Use MTrackJ Plugin

**Fig. 2:**
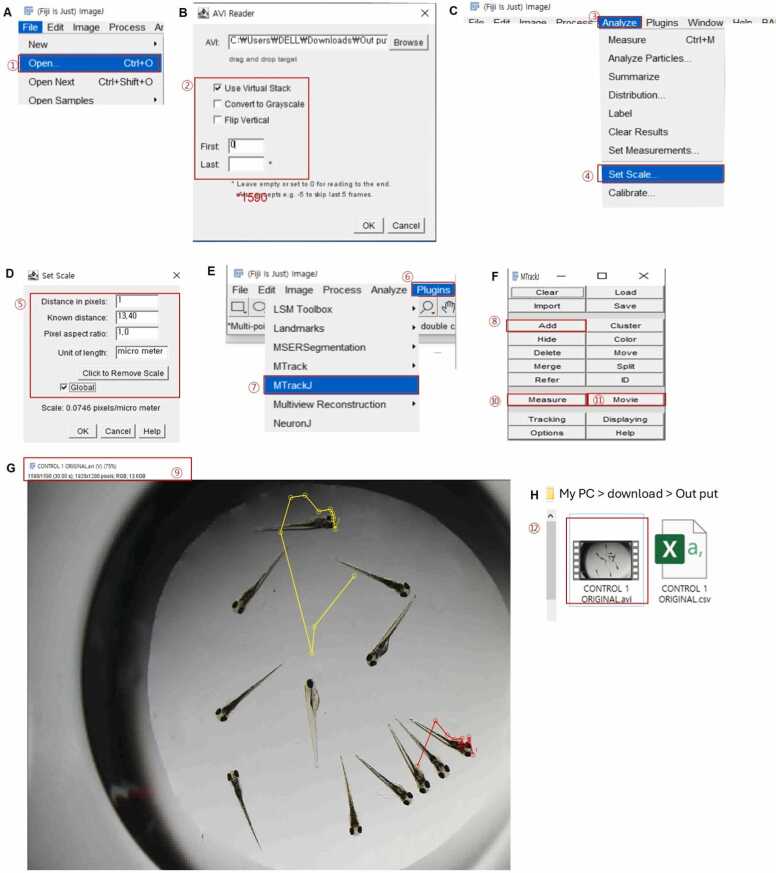
Method for locomotive activity test using ImageJ1.56fFiji “MTrackJ” plugin, showing an example for manual tracking by touch response test and agitation challenge recorded videos. This method would be used to quantify the locomotive activity of behavior recorded videos as distance in x-axis, distance in y-axis, length of track, velocity, angle of turn, and others, and to generate visible swim videos paths with assigned numbers (track identification number: TID) for multiple zebrafish embryos in a same video. (A) Path to open the video in Fiji ImageJ. (B) AVI Reader window box for selecting the stack range of the video to be opened, 1 stack = 0.02 s. (C) Path for opening the MTrackJ plugin. (D) MTrackJ window box. (E) MTrackJ in *.avi format video. (F-H) The output of behavior tracked video in *.avi format and behavior measurements in *.csv format. Red color numbering denotes the sequence of steps by which the method of the tracking.

The MTrackJ plugin of ImageJ1.54fFiji (NIH) can also generate distance traveled, velocity, and visible swim path. First, open a *.avi format video file, then AVI Reader window will pop up (Fig. 2A and B). To select the distance and analyze from a video, check the visual stack to be used. Put 0 or 1 in the first stack, and put the time length value converted to stacks of the time required video to decide the last stack of the video (Fig. 2B). The time length of the video can be obtained automatically from the video file opened in ImageJ1.54fFiji and seen at the top left corner. Videos are normally opened as stacks using ImageJ1.54fFiji. Note that in ImageJ1.56fFiji, 1 stack is set to 0.02 s as a default. To get a movement length as a pixel from a video file, put the scale bar length obtained from the magnification at which the video was shot into Set Scale to obtain the length for 1 pixel (length calibration in pixel, Fig. 1E). And then, open the MTrackJ plugin (Fig. 2C), then the reformatted file from the video file window (Fig. 2E) would pop up. Each one is individually chosen by mouse click and selected from the Add > Measure in the MTrackJ window (Fig. 2D). Then, a *.csv format file would appear where the locomotive activity is quantified as distance in x-axis, distance in y-axis, length of track, velocity, angle of turn, and so on. Thereafter, clicking Movie in the MTrackJ window (Fig. 2D) would generate a video of swim paths with an assigned track identification number (TID) to each embryo in the video (Fig. 2F). It should be noted that one separate TID is assigned to each embryo without overlapping with other embryos when tracks are created using the “Add track” function in MTrackJ plugin. Then, the TID of each embryo will be assigned automatically.

## CONCLUDING REMARKS

The VirtualDub2 (SourceForge) is frequently used as open-source software for converting video recording from *.mp4 to *.avi format due to the limitation of the supported video format algorithms in ImageJ (Kurnia et al., 2023). Fiji (NIH) is a distribution of ImageJ, a well-known with long history open-source program designed for biological-image analysis. Fiji enables quick prototyping of image-processing algorithms by combining robust software libraries with various scripting languages using contemporary software engineering techniques (Schindelin et al., 2012). In video tracking, Fiji helps users to properly interpret information extracted from trajectories by avoiding the need for complicated tools and offering user-friendly data analysis schemes (López et al., 2023). In this technique, we utilized Fiji's "Manual Tracking" and "MTrackJ" plugins to meticulously track locomotive activity of zebrafish embryos. This approach assesses thigmotaxis caused by anxiety and the deleterious influence on neurotransmission due to toxicant exposure in the touch response test (Paredes-Zúñiga et al., 2019). This was also utilized to assess zebrafish embryos' ability to retain postural stability after they lost balance during the agitation test. This approach might readily be adapted for use in other modeling systems. However, there is no user manual for the application provided, which makes initial use difficult. Therefore, we would like to provide a kind of manual for supporting new users.

## CRediT authorship contribution statement

**Ranasinghe Thilini:** Writing – original draft. **Cha Seon-Heui:** Writing – review & editing, Investigation, Funding acquisition, Conceptualization.

## AUTHOR CONTRIBUTIONS

T. Ranasinghe drafted the main body section. S.-H. Cha conceived for overall concept, provided funding, wrote draft, and final manuscript.

## DECLARATION OF COMPETING INTERESTS

The authors declare that they have no known competing financial interests or personal relationships that could have appeared to influence the work reported in this paper.
