# Supplementary material for: Open-source software utilization for zebrafish embryos behavior test
Source: Mol Cells. 2025 Apr 25;48(6):100221. doi: 10.1016/j.mocell.2025.100221 (PMC12140941; doi:10.1016/j.mocell.2025.100221)
Supplement: Supplementary file 1 — Supplementary material [file mmc1.docx]

# **Open-source software utilization for zebrafish embryos behavior test**

Thilini Ranasinghe, Seon-Heui Cha

# **Supplementary Methods**

### *Conversion of video format*

The *.mp4 format video is initially obtained from a microscope recordings, is subjected to video format conversion using VirtualDub2 (SourceForge) as shown in **Supplementary Figure. 1.** Batch Wizard and VirtualDub2 Job Control windows are mainly used for this purpose. The generated video is created in gigabyte size. The video converted to *.avi format by VirtualDub2 (SourceForge) should be used when working with ImageJ1.54f Fiji (NIH).

### *Calculating the meaningful quantified locomotive activity with displayed values*

The quantified locomotive activity output of *.csv format files does not give meaningful values until it is processed. The processing method is displayed in an example in **Supplementary Figure. 2**, where the addition/sum of values is taken vertically under each variable leaving the first recorded point. This method applies to both the first and second methods of manual tracking.

**Supplementary Results**


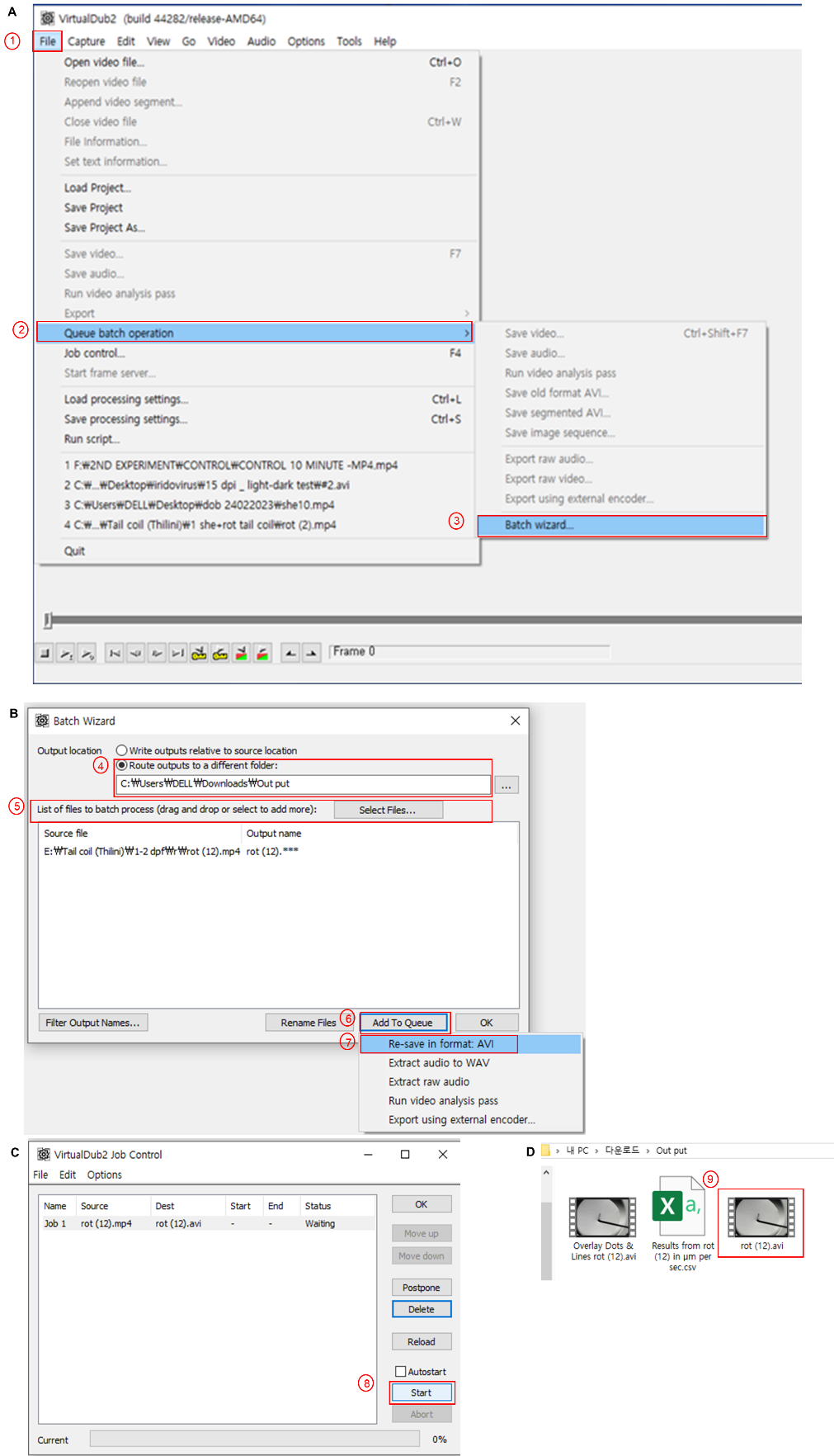


### **Supplementary Figure. 1. Conversion of behavior recorded videos to ImageJ1.54fFiji usable format using VirtualDub2.** (A) Opening the Batch wizard queque batch operation. (B) Assigning route output of tracked video, selecting the path of the video to be used in behavior tracking in *.mp4 format. Adding to queue to be re-saved in *.avi format. (C) VirtualDub2 Job Control window box showing progress of conversion from *.mp4 to *.avi. (D) Video out put in *.avi format. Red color numbering denotes the sequence of steps by which the extension *.mp4 format video file would be converted to *.avi format video file.


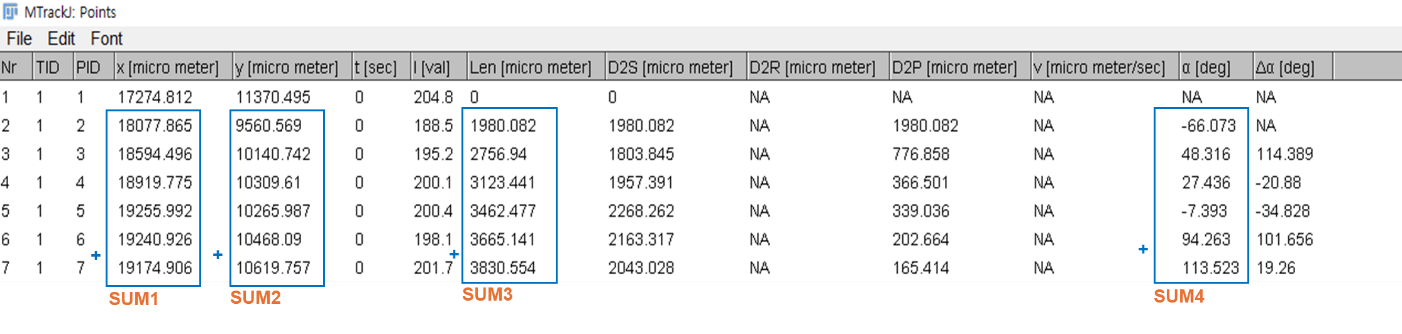


### **Supplementary Figure. 2. Quantification of locomotive activity by second method for manual tracking using ImageJ1.54fFiji.** This output file could be saved as an extension *.csv file. Locomotive activity of behavior recorded videos were quantified as SUM1:distance in the x-axis, SUM2:distance in the y-axis, SUM3:length of track, SUM4:angle of turn and others, and to generate videos with visible swim paths with separate assigned numbers (track identification number:TID) for multiple zebrafish embryos in the same video. Velocity can be obtain from SUM3÷video length in sec.
